# simDP: Sim-to-Real Transfer with Shared Action Spaces

**DOI:** 10.3390/s26134079

**Published:** 2026-06-27

**Authors:** Chanhyuk Jung, Jongbin Choi, Sungkeun Yoo, Byoung Chul Ko

**Affiliations:** 1 Department of Computer Engineering, Keimyung University, Daegu 42601, Republic of Korea; seagullcjung@gmail.com; 2 Department of Robot Engineering, Keimyung University, Daegu 42601, Republic of Korea; walt0504@stu.kmu.ac.kr (J.C.); skyoo@kmu.ac.kr (S.Y.)

**Keywords:** sim-to-real, shared action space, real-world robotics, diffusion policy, robotic manipulation

## Abstract

In this paper, we propose simDP, a sim-to-real transfer framework that enables diffusion policies trained in simulations for the efficient deployment on real-world robots. The key idea is to reduce the sim-to-real gap by aligning the action and observation spaces between simulation and reality. Specifically, we reformulate the action space using end-effector pose and binary gripper state, which can be shared between simulated and physical robots. In addition, we use camera-based visual observations as the primary sensing modality in both domains and train a real-world observation encoder to align with the latent representation learned in simulation. This design allows the action decoder trained in simulation to be reused in the real-world with minimal modification. We evaluated simDP on object manipulation tasks derived from the MimicGen benchmark and show that a simulation-trained diffusion decoder, when combined with a real-world adapted observation encoder, achieves task completion performance similar to and in some cases better than diffusion policies trained only on limited real-world data. These results, obtained across four manipulation tasks in a calibrated real-world transfer setting, suggest that reusing a simulation-trained action decoder with lightweight real-world encoder adaptation provides an effective strategy for controlled sim-to-real transfer, while broader evaluation across diverse tasks, environments, and robot embodiments remains an important direction for future work.

## 1. Introduction

Recent advances in large language models have shown that scaling model capacity together with high-quality data and improved training strategies can produce remarkable generalization ability across a wide range of tasks. A similar trend has emerged in multimodal learning, where vision–language models extend this paradigm by jointly processing textual and visual inputs and enabling reasoning over both modalities. Building on this progress, robotics has begun to adopt analogous formulations in the form of vision–language action (VLA) models, which aim to generate robot actions conditioned on language instructions and sensory observations.

Despite this promise, robotic learning remains fundamentally more difficult to scale than language or vision learning. Unlike text and image data, which can often be collected from large online corpora, robot manipulation data typically require teleoperation, physical hardware, carefully designed task environments, and repeated human intervention. As a result, the collection of large-scale real-world robotic datasets is expensive, slow, and difficult to parallelize [1]. In addition, directly training physical robots introduces practical challenges such as safety risks, hardware wear, nontrivial environment resets, and difficulty obtaining reliable reward signals or task-state annotations.

These challenges make simulation an attractive alternative for robot policy learning. In simulated environments, task configurations can be generated more easily, environment resets can be automated, and privileged state information can be accessed directly for supervision or evaluation. Simulation also enables scalable data collection without requiring a proportional increase in the number of physical robots and experimental setups. Therefore, simulation has become an increasingly important platform for training data-intensive visuomotor policies, including diffusion-based policies and emerging VLA robotic systems.

However, the benefits of simulation can only be fully realized if the policies learned in simulations can be transferred effectively to real robots. This remains difficult because of the well-known sim-to-real gap. A major source of this gap arises from the discrepancy between simulated and real-world observations: rendered images in simulation differ from real camera images in terms of appearance, lighting, texture, noise, and sensor artifacts. Another important source of mismatch lies in the action space. Many robot policies are defined using robot-specific control variables, such as joint configurations, which do not naturally transfer across different embodiments or between simulated and physical systems. Therefore, successful sim-to-real transfer requires both robust adaptation to differences in observations and an action representation that can be shared consistently across environments.

In this paper, we propose simDP, a sim-to-real transfer framework for diffusion policies [2] that explicitly addresses both issues. We decompose the diffusion policy (DP) into an observation encoder and an action decoder. To bridge the action–space gap, we define actions using the end-effector pose and binary gripper state, which provide a shared and robot-agnostic control representation across simulation and real-world systems. To bridge the observation–space gap, we use camera-based visual observations as the primary sensing modality in both simulation and the real-world. We then train a real-world observation encoder to align its latent output with the latent space learned in simulation, while keeping the simulation-trained action decoder fixed. This design allows the policy’s action-generation module to be learned entirely in simulation and then reused in the real-world by adapting only the observation encoder through latent-space alignment.

We evaluate simDP on object manipulation tasks derived from the MimicGen benchmark [3] and test transfer performance on a physical robot. Experimental results show that our method achieves performance similar to, and in some cases better than, diffusion policies trained directly on limited real-world demonstrations. These findings indicate that aligning shared action representations and latent observation spaces is an effective strategy for sim-to-real transfer in diffusion-based robotic learning. Our contributions are summarized as follows:

- We propose simDP, a sim-to-real transfer framework for diffusion policies based on a shared action space and latent observation alignment.
- We show that end-effector-based actions enable the simulation-trained action decoder to be transferred across simulation and real-world robotic setups.
- We employ camera-based observations and MimicGen-based real-world manipulation tasks to demonstrate that simDP achieves similar or superior performance compared with diffusion policies trained on limited real-world data.

## 2. Related Work

### 2.1. Sim-to-Real Transfer for Robot Manipulation

Sim-to-real transfer, which leverages simulation during training, has long been studied as a practical strategy for reducing the cost of real-world robot data collection. Its primary challenge is the discrepancies between simulation and reality in visual appearance, physical dynamics, and embodiment-specific execution conditions. One common approach to reducing these gaps is to use modalities such as depth, point clouds, or tactile feedback, which are often less sensitive to appearance variation than RGB camera observations. The use of such modalities can improve robustness. However, they may discard fine-grained visual information that is important for object discrimination and precise manipulation. This limitation is particularly relevant when the robot must distinguish objects with similar shapes but different appearances, or when manipulation depends on high-resolution visual cues.

Several studies [4,5,6,7,8,9] have identified appearance mismatch as one of the primary sources of transfer failure for RGB-based policies, motivating the continued search for more robust observation representations and transfer mechanisms. A widely used solution for RGB-based sim-to-real transfer is domain randomization. Domain randomization was established as an effective sim-to-real transfer technique in [7,10,11], which showed that randomizing textures, lighting, materials, camera poses, and other rendering factors enables transfer from simulated RGB images to real-world robotic control. Later work extended this idea by adaptively optimizing the randomization distribution. For example, Bayesian domain randomization (BayRn) [9] adjusts simulator parameter distributions using sparse real-world feedback, improving transfer efficiency when the target domain is not well characterized in advance. Domain randomization relies on making the distributions of simulation observations wide enough to encompass images that can be observed in the real-world. In contrast, our work keeps the simulation-trained decoder fixed and adapts only the real-world encoder so that real observations are mapped into the latent space that this decoder already expects, focusing the adaptation on reliable real-world decoding rather than on widening the simulation observation distribution.

Other works instead reduce the gap at the image level by transforming real-world RGB images so that their appearance resembles that of simulation scenes. Randomized-to-Canonical Adaptation Networks (RCAN) [12] learn an image-translation module that canonicalizes both domain-randomized simulation images and real-world images into a common non-randomized simulation appearance, enabling zero-shot transfer of RL policies trained only in simulation. Because this module is prepended to an existing policy, the approach is broadly applicable to many visuomotor policies. However, it introduces an additional image-generation stage: real-world observations must first be translated into canonical images before they can be encoded into features usable by the action decoder. In contrast, our work directly retrains the observation encoder so that real-world observations are mapped into features compatible with the simulation-trained action decoder, bypassing the need for an intermediate image-generation model (see Section 2.4 for further discussion of this adaptation mechanism).

Another approach is to improve the realism of the simulator itself. Instead of relying only on randomized mesh-based rendering, recent neural rendering approaches attempt to reconstruct real scenes more faithfully, thereby directly narrowing the visual gap. For example, SplatSim [13] uses Gaussian-splatting-based photorealistic scene representations to support RGB camera-based manipulation transfer and realistic evaluation. These methods improve visual fidelity, but they often require additional reconstruction pipelines or scene-specific preparation. In contrast, our work does not attempt to fully recreate the target scene; rather, it reduces the transfer gap by combining a shared action representation with latent observation alignment between simulation and reality.

### 2.2. Diffusion-Based Visuomotor Policies

Diffusion models have recently emerged as a powerful framework for robot manipulation [2,14,15,16,17] because they can model complex and multimodal action distributions while maintaining stable optimization behavior. DP [2] formulates visuomotor control as conditional denoising over action trajectories, where the model iteratively refines a noisy action sequence into a valid future plan conditioned on observations. This formulation has shown strong performance across diverse robot manipulation tasks and has become a representative baseline for imitation-based visuomotor learning. The present work builds directly on this line of work by decomposing DP into an observation encoder and an action decoder, treating the two components as separable modules amenable to domain-specific adaptation.

The relevance of diffusion models to our setting is twofold. First, their ability to represent multimodal action distributions makes them especially effective for manipulation tasks with diverse valid strategies. Second, diffusion-based policies are highly data-driven, which makes simulation an attractive source of scalable training data. MimicGen [3] further supports this direction by automatically synthesizing over 50,000 demonstrations across 18 tasks from a relatively small number of human demonstrations, showing that large-scale imitation datasets can be generated efficiently for robot learning. However, most prior DP studies focus on training and evaluation within a fixed embodiment or rely on real-world data for deployment, leaving open the question of how to reuse a simulation-trained DP on physical robots with minimal real-world adaptation. Recent work has also explored flow-based visuomotor policies beyond diffusion-based denoising. In particular, shifted flow policy (SFP) introduces uncertainty-aware time reparameterization to improve action generation over future horizons, highlighting that temporal design is another important factor in generative visuomotor policy learning [18].

Compared with prior work, our method is distinguished not by the introduction of a new diffusion backbone but by the proposal of a new transfer mechanism tailored to diffusion policies. Specifically, we reuse the simulation-trained action decoder and adapt only the real-world observation encoder so that its latent output matches the latent distribution expected by the decoder. This modular design directly reflects the central premise of the paper: the action generation process can be learned in simulation if the actions and observations are aligned appropriately across domains.

### 2.3. Shared Action Spaces and Cross-Embodiment Transfer

In addition to observation mismatch, how actions are represented strongly affects robot transfer. Many manipulation policies operate in robot-specific action spaces, such as joint positions or low-level motor commands, which makes transfer across different embodiments difficult. This issue is directly relevant to the present work, where the action space is redefined using end-effector pose and binary gripper state so that the same policy decoder can be reused in both simulation and real-world systems.

Recent large-scale robot learning work has highlighted the importance of using shared or coarsely aligned action interfaces across heterogeneous robots. Open X-Embodiment [1] aggregated data from many robots and institutions into a standardized framework and showed that cross-robot transfer can improve manipulation capability when diverse embodiments are brought into a common data interface. Building on this, Octo [19] demonstrated that a generalist robot policy can be pretrained on 800k trajectories and later fine-tuned to new setups with different sensory inputs and action spaces. These studies suggest that action–space standardization is a key ingredient for scalable robot learning across embodiments and directly motivate our choice to define actions in a robot-agnostic end-effector space.

A complementary line of work demonstrates the scalability of policies trained on large, diverse real-world corpora. RT-1 [20] demonstrated that large policies trained on diverse real-world demonstrations can generalize across multiple tasks and robot embodiments, but at the substantial cost of collecting and curating such datasets. Our work is complementary to this direction: rather than scaling real-world data collection, we study how a simulation-trained DP can be transferred to reality through latent observation alignment, treating data efficiency as the primary design objective.

### 2.4. Relation to Representation Alignment, Modular Imitation Learning, and Fine-Tuning

simDP is also adjacent to three transfer paradigms not covered above. Representation-alignment methods [21,22] reduce the sim-to-real gap by adding an auxiliary objective that encourages feature distributions from different domains to become similar, for example through adversarial domain confusion or correlation-based alignment. In contrast, simDP does not introduce an explicit distribution-matching objective. Instead, the real-world encoder is trained only through the action-prediction loss induced by the frozen decoder, so the latent representation is shaped by what the decoder requires for action generation rather than by direct similarity to the simulation feature distribution.

Modular imitation learning and sim-to-real policy transfer methods also decompose a policy into perception and control-related components. For example, Müller et al. [23] train a driving policy in simulation using an abstract semantic interface and transfer it to the real world through a modular architecture consisting of perception, policy, and low-level control. simDP follows a related modular philosophy, but adopts a stricter encoder–decoder separation: the decoder is permanently frozen after simulation training, and all real-world adaptation capacity is confined to the encoder. This design preserves the simulation-trained action prior exactly.

Finally, sim-to-real fine-tuning updates part or all of a simulation-trained policy using real-world data [24]. Such fine-tuning is an effective transfer strategy, but because policy parameters are updated, it does not preserve the simulation-trained action prior by construction. In contrast, simDP never updates the decoder and learns only the observation-to-latent mapping for real-world inputs. The common distinction is that adaptation in simDP is driven by a downstream action loss rather than by a distribution-matching criterion, and is confined entirely to the encoder under a frozen decoder.

## 3. Method

**Figure 1:**
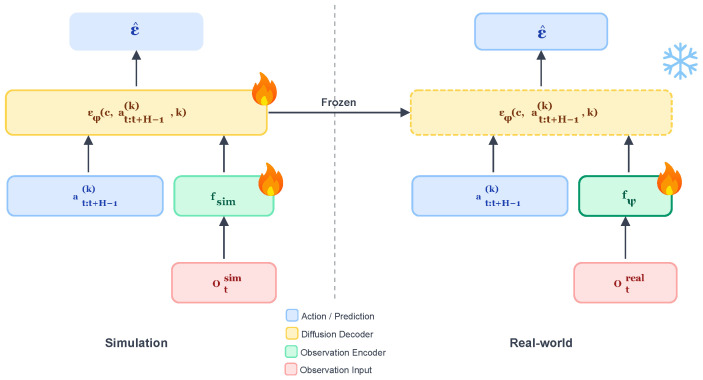
Overview of simDP. (**Left**) In the simulation stage, the policy is trained using a shared end-effector action representation and a simulation observation encoder $f_{\mathrm{sim}}$. (**Right**) In the real-world adaptation stage, the diffusion decoder $\epsilon_{\phi }^{*}$ is frozen and only the real observation encoder $f_{\psi }$ is optimized so that its latent output becomes compatible with the frozen decoder. Here, $a_{t:t+H-1}^{\left(k\right)}$ denotes the noised action chunk at diffusion step *k*, and $\hat{\epsilon }$ denotes the predicted noise.

The goal of simDP is to transfer a DP learned in simulation to a real robot while minimizing the amount of real-world policy retraining. Our approach is based on two design choices as shown in Figure 1. First, we define a shared action representation that is valid in both simulation and real-world environments. Second, we decompose the policy into an observation encoder and an action decoder, and adapt only the real-world observation encoder while keeping the simulation-trained decoder fixed. This design allows the action-generation process to be learned from abundant simulation data and reused in the real-world through latent-space compatibility.

Specifically, instead of using robot-specific joint commands as actions, we represent each action by the end-effector position, end-effector orientation, and gripper state. Let

(1) $$a_{t}=\left[p_{t},r_{t},g_{t}\right],$$

where $p_{t}\in R^{3}$ denotes the Cartesian position of the end-effector, $r_{t}\in R^{6}$ denotes the end-effector orientation represented using a continuous 6D rotation representation [25], and $g_{t}\in \left\{0,1\right\}$ denotes the binary gripper state, where we assume a parallel-jaw gripper with a binary open/close command. Dexterous manipulation with multi-fingered hands is outside the scope of this shared action representation. This representation is robot-agnostic in the sense that it describes task-relevant end-effector motion independently of the specific joint configuration of a given robot. The corresponding joint angles for each embodiment are recovered through inverse kinematics at execution time.

Let the observation at time *t* be denoted by $o_{t}$ and consist of the visual input and proprioceptive state. Following DP [2], we predict an *H*-step future action chunk

(2) $$a_{t:t+H-1}=\left(a_{t},a_{t+1},\ldots ,a_{t+H-1}\right).$$

During training, a diffusion step k∈{1,…,K} and Gaussian noise ϵ∼N(0,I) are sampled, and the clean action chunk is corrupted as

(3) $$a_{t:t+H-1}^{\left(k\right)}=\sqrt{{\bar{\alpha }}_{k}}\;a_{t:t+H-1}+\sqrt{1-{\bar{\alpha }}_{k}}\;\epsilon ,$$

where ${\bar{\alpha }}_{k}$ is the cumulative noise schedule coefficient at step *k*. Given the simulation observation encoder $f_{\mathrm{sim}}$ and noise prediction network $\epsilon_{\phi }$, the simulation-stage training objective is

(4) $$L_{\mathrm{sim}}=E_{(o_{t},a_{t:t+H-1}),\;\epsilon ,\;k}\left[{∥\epsilon -\epsilon_{\phi }\;\left(f_{\mathrm{sim}}\left(o_{t}\right),a_{t:t+H-1}^{\left(k\right)},k\right)∥}_{2}^{2}\right],$$

In our implementation, $f_{\mathrm{sim}}$ is a data-augmented ResNet-18 encoder [26] and $\epsilon_{\phi }$ is a 1D U-Net [27] following the standard DP architecture.

### 3.1. Real-World Observation Encoder Adaptation

Although the shared end-effector action representation reduces the embodiment gap, the visual observation distributions of the simulation and the real-world still differ significantly. Differences in texture, lighting, camera characteristics, and robot appearance prevent the simulation observation encoder from generalizing directly to real images. To address this issue, we replace the simulation encoder with a real-world observation encoder $f_{\psi }$ and adapt only this encoder using real-world demonstrations, while keeping the simulation-trained noise prediction network fixed.

Importantly, our objective does not explicitly force the real encoder to match the simulation latent distribution in a point wise manner. Instead, it trains the real encoder to produce latent features that are compatible with the frozen decoder. Given real-world demonstrations, the real encoder is optimized using

(5) $$L_{\mathrm{real}}=E_{(o_{t}^{\mathrm{real}},a_{t:t+H-1}^{\mathrm{real}}),\;\epsilon ,\;k}\left[{∥\epsilon -\epsilon_{\phi }^{*}\;\left(f_{\psi }\left(o_{t}^{\mathrm{real}}\right),a_{t:t+H-1}^{(k),\mathrm{real}},k\right)∥}_{2}^{2}\right],$$

where $\epsilon_{\phi }^{*}$ denotes the frozen simulation-trained decoder and only the encoder parameters ψ are updated. This objective encourages the real-world encoder to map observations into a decoder-compatible latent space, thereby enabling the simulation-trained action generator to operate on real-world inputs.

To make the shared action representation valid across domains, we also calibrate the simulation and real-world setups so that the workspace scale, camera viewpoint, and end-effector coordinate convention are relatively similar.

### 3.2. Policy Rollout

At inference time, the real-world observation $o_{t}^{\mathrm{real}}$ is first encoded as

(6) $$c_{t}=f_{\psi }\left(o_{t}^{\mathrm{real}}\right).$$

Starting from Gaussian noise $a_{t:t+H-1}^{\left(K\right)}∼N\left(0,I\right)$, the frozen decoder iteratively denoises the action chunk according to the reverse diffusion process,

(7) $$a_{t:t+H-1}^{\left(k-1\right)}=\Phi_{k}\left(a_{t:t+H-1}^{\left(k\right)},\epsilon_{\phi }^{*}\left(c_{t},a_{t:t+H-1}^{\left(k\right)},k\right)\right),\;k=K,\ldots ,1,$$

where $\Phi_{k}\left(\cdot \right)$ denotes the denoising process of the diffusion model. After the denoising process is completed, the first action in the predicted chunk is executed on the robot, and the process is repeated in a receding-horizon manner.

This rollout strategy preserves the action-generation capability learned in simulation while adapting only the observation interface to the real-world. As a result, simDP enables efficient sim-to-real transfer of diffusion-based manipulation policies without the need to retrain the full policy on large real-world datasets.

## 4. Experiments

This section presents the evaluation of simDP on real-world manipulation tasks. We recreated the tasks to match the scales and general shapes of objects in the simulations and calibrated our robot arm to closely match the movement of the robot arm in simulations by using the same control frequency and tuning the Proportional-Integral-Derivative (PID) values of the motors. At execution time, each commanded end-effector pose is mapped to joint angles by the robot’s inverse kinematics solver. We restrict task execution to a workspace region in which the inverse kinematics solution is well-defined and away from kinematic singularities, so that small changes in the commanded end-effector pose produce continuous changes in joint configuration and the shared action space behaves consistently across simulation and reality.

The main objective is to verify whether a DP trained in simulation can be effectively transferred to a physical robot through the proposed shared action representation and decoder-compatible latent observation alignment. We further examine whether the proposed transfer strategy can outperform policies trained directly on limited real-world data. To provide a broader empirical comparison, we include several representative policy baselines: DP [2], flow matching policy (FMP) [28], and action chunking transformer (ACT) [29].

### 4.1. Dataset

We evaluated simDP on four manipulation tasks from MimicGen [3]: *Stack Three*, *Three Piece Assembly*, *Threading*, and *Square*. These tasks were selected to cover a range of manipulation difficulty levels. *Stack Three* represents a relatively simple sequential pick-and-place task, whereas *Three Piece Assembly* requires precise geometric alignment and contact-rich insertion. *Threading* introduces fine-grained manipulation with narrow tolerance. *Square* is the inverse of *Threading* where a square block with a hole is inserted into a stationary pole. Together, these tasks provide a comprehensive evaluation of both basic and challenging contact-rich manipulation settings.

For the simulation training, we used the corresponding MimicGen demonstrations for each task. For the real-world transfer, we followed the proposed simDP framework and collected 50 demonstrations per task to train the real-world observation encoder while keeping the simulation-trained decoder frozen.

Reconstructing each MimicGen task in the real world is substantially more demanding than evaluating in simulation: object scales and shapes, scene layout, camera viewpoint, and the end-effector coordinate convention must all be matched to the simulator, and the robot arm must be calibrated as described above. Because every rollout additionally requires human-supervised resets and safety monitoring, large-scale real-world evaluation is costly, and prior real-robot manipulation studies typically report results on a comparable number of tasks and trials.

### 4.2. Real-World Results

Table 1 reports the real-world normalized task completion (NTC) results on the four evaluation tasks. We reconstructed the MimicGen task environments on a real-world robot platform and evaluated all methods using the same task objectives as in the original MimicGen tasks. The main evaluation metric is NTC, measured over 15 randomly initialized real-world rollouts. Because policies often complete only part of a task, we quantify progress using subtask completion milestones and normalize the resulting score to the range [0, 1], where 1.0 indicates that all subtasks required for full task completion are successfully achieved. Each value in Table 1 is reported as the average over the 15 rollouts. All methods were evaluated under the same observation setting, evaluation protocol, action chunk execution pipeline, and success criterion to ensure a fair comparison.

The results in Table 1 indicate that simDP consistently outperforms both the implicit policies based on generative models and the explicit policies based on the transformer architecture on all tasks. Compared with DP, the performance gain is relatively moderate on *Stack Three*, *Threading*, and *Square*, but increases substantially on *Three Piece Assembly*. This trend suggests that the benefit of simulation pretraining becomes more pronounced as the task becomes more contact-sensitive or execution-critical.

In particular, the strong performance on *Three Piece Assembly* indicates that the proposed shared action representation and latent observation adaptation help preserve precise action generation under substantial visual and embodiment mismatch. Overall, these results support the central claim of this paper: a simulation-trained diffusion decoder can be effectively reused in the real-world when the observation interface is properly adapted and the action space is shared across domains.

A notable pattern in Table 1 is that the advantage of simDP becomes larger on *Three Piece Assembly* than on the remaining three tasks.

This difference is meaningful because Three Piece Assembly requires not only correct reaching and grasping, but also stable geometric alignment and contact-sensitive insertion. In such tasks, small observation errors can easily accumulate into large execution failures. The stronger performance of simDP on this task therefore suggests that reusing a simulation-trained decoder provides a more reliable action prior when the downstream behavior requires precise multi-stage coordination. This observation also supports our design choice of separating action generation from observation adaptation, since the decoder appears to preserve task structure learned from simulation even under real-world visual mismatch.

### 4.3. Sim-to-Real Transfer Ablations

To verify the effectiveness of the proposed transfer mechanism, we compared simDP—where a simulation-trained decoder is reused and only the real-world observation encoder is adapted—with four alternative strategies: (1) zero-shot sim-to-real transfer, (2) training from scratch on real-world data, (3) the proposed decoder reuse with encoder adaptation, and (4) DP (sim-trained, sim-evaluated) as a simulation-only baseline. This fourth strategy is included solely as a simulation-only reference point, not an upper bound on real-world performance. It indicates the decoder’s completion level in its native simulation domain before transfer. simDP, operating on real-world observations, recovers and slightly exceeds this level, since its encoder is adapted on task-specific demonstrations of the evaluation scenes. For a more fine-grained evaluation, we measured the average normalized task completion score for each strategy. Because exact continuous task completion is difficult to define, we used subtask completion milestones to compute a coarse but consistent progress score in the range [0, 1], as reported in Table 2. Because the *Threading* task exhibited substantially higher rollout variance and longer execution time during repeated real-world evaluation, we report the ablation comparison on *Stack Three* and *Three Piece Assembly*, which provide a more stable basis for controlled strategy comparison.

As expected, direct zero-shot transfer leads to catastrophic failure in which the model fails to understand the scene because of the huge disparity between the simulation and real-world observations. Training DP from scratch enables the model to complete some subtasks, but because of the limited quantity and quality of the demonstrations, real-world performance is degraded with respect to the simulation-only trained DP’s performance. In contrast, simDP can apply the skills learned from simulations in the real-world, outperforming DP trained only on real-world demonstrations. This result suggests that utilizing the simulation-trained decoder is beneficial, since it already captures a strong action-generation prior learned from a large simulation dataset.

### 4.4. Shared Observation Space

The design goal of simDP is to align the observation space and action space between simulation and real-world environments. While the shared action representation can be transferred relatively naturally across domains, visual observations remain substantially different because of changes in object appearance, lighting, camera characteristics, and scene composition. To address this gap, we train the real-world observation encoder to produce latent features that are compatible with the simulation-trained decoder.

To qualitatively examine whether the simulation and real-world encoders produce compatible latent representations, we visualize their embeddings using t-SNE [30]. Before applying t-SNE, we first reduced the latent features to 32 dimensions using truncated singular value decomposition (SVD) to reduce the sparsity of the embeddings computed by the observation encoders to prevent t-SNE from collapsing. This can happen as sparse embeddings have lower distances between each other when they are in fact different from one another. For better visibility, we limited the number of samples to 10,000 simulation embeddings and 2500 real-world embeddings, while roughly retaining the ratio between the number of simulation and real-world samples. We fixed the random seed to 42, and set perplexity to 30.0 to run t-SNE on the 32 dimension embeddings.

**Figure 2:**
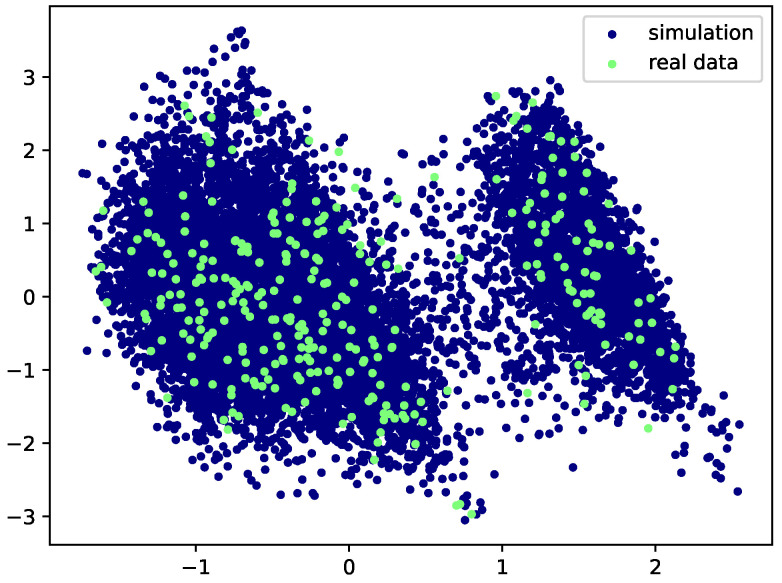
t-SNE visualization of latent embeddings produced by the simulation observation encoder and the adapted real-world observation encoder. Before applying t-SNE, the latent features were reduced to 32 dimensions using truncated SVD. We used 10,000 simulation samples and 2500 real-world samples, with a perplexity of 30.0 and a random seed of 42. The two domains show substantial qualitative overlap, suggesting approximate latent-space compatibility, while the simulation embeddings appear denser because more simulation data were available.

As shown in Figure 2, the embeddings from the two domains exhibit substantial qualitative overlap in the 2D visualization, suggesting that the adapted real-world encoder produces latent features that are broadly compatible with those of the simulation encoder. We also observe that the simulation embeddings appear denser and more widely distributed than the real-world embeddings, which is likely related to the larger amount of simulation data used during training. However, because t-SNE is a nonlinear visualization tool that may distort global geometry, this result should be interpreted only as qualitative evidence of latent-space compatibility rather than as a strict proof of distributional alignment.

### 4.5. Real-World Data Efficiency

One of the main motivations of simDP is to reduce dependence on large-scale real-world demonstrations. To evaluate this aspect, we compared simDP and DP trained from scratch with varying numbers of real-world demonstrations. For the data-efficiency study, we focus on *Stack Three* and *Three Piece Assembly*, since these two tasks allow a clearer comparison of transfer trends under different amounts of real-world data while keeping repeated real-world evaluation manageable.

Table 3 shows that simDP outperforms DP trained from scratch when the same amount of real-world data is used. Each entry is reported as the average over 15 real-world rollouts. The performance gap is especially large in the low-data regime, where the simulation-trained decoder provides a strong prior and the real-world encoder requires only modest supervision to adapt to the target domain. As the amount of real-world data increases, the gap gradually narrows, but when a certain amount of data is available, simDP jumps in performance and steadily increases as more data is available. This behavior is desirable because the proposed method is intended for precisely the setting where collecting real-world robot data is expensive and limited.

These data-efficiency results also provide an important practical implication. In many real-world robotics settings, collecting demonstrations is not only expensive but also slow because each rollout requires human supervision, environment reset, and safety monitoring. Under this constraint, a transfer method is most useful when it can achieve acceptable performance before a large real-world dataset becomes available. The results in Table 3 indicate that simDP is particularly effective in this regime. Rather than learning the full policy from scratch, the method only needs to adapt the observation interface while preserving the action-generation capability already acquired in simulation. This makes simDP attractive for applications where real-world data are scarce, task setup is costly, or rapid deployment is required.

### 4.6. Qualitative Analysis

**Figure 3:**
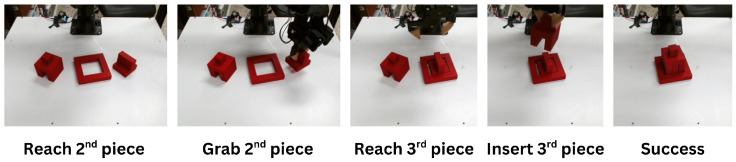
Representative real-world rollout of simDP on the *Three Piece Assembly* task. The sequence illustrates the successful execution of multi-stage manipulation, including grasping, alignment, insertion, and final assembly completion.

Figure 3 presents representative real-world rollouts of simDP on the *Three Piece Assembly* task. This qualitative sequence illustrates that the proposed policy can successfully execute multiple subtasks in the correct order, including grasping, alignment, insertion, and final assembly. This qualitative evidence complements the quantitative results by showing that the performance gain of simDP is associated with stable multi-stage behavior rather than isolated short-horizon successes.

## 5. Conclusions

In this paper, we proposed simDP, a sim-to-real transfer framework for diffusion-based robotic manipulation. The key idea of simDP is to reduce the sim-to-real gap from two perspectives: a shared action representation and real-world observation adaptation. Specifically, we define actions using end-effector pose and binary gripper state so that the same action space can be used in both simulation and real environments. In addition, we decompose the diffusion policy into an observation encoder and an action decoder, and adapt only the real-world encoder while keeping the simulation-trained decoder fixed. This design enables the policy to reuse the action-generation capability learned from scalable simulation data while requiring only limited real-world adaptation of the observation encoder.

Experimental results on four MimicGen-derived real-world manipulation tasks, evaluated in a calibrated real-world transfer setting, showed that simDP achieved consistently strong transfer performance and outperformed representative baselines within the considered evaluation setting. The ablation studies further indicated that reusing the simulation-trained decoder together with real-world observation encoder adaptation was more effective than direct zero-shot transfer or training from scratch on limited real-world data. These results suggest that effective sim-to-real transfer can be achieved without retraining the full diffusion policy in the real world, provided that the action space is shared and the real-world observation interface is properly adapted. We regard these findings as evidence for a practical and data-efficient transfer mechanism, while broader evaluation across more diverse tasks, environments, and robot embodiments remains an important direction for future work.

Despite these promising results, several limitations remain. First, the current real-world evaluation reports mean normalized task completion scores, but per-rollout variance was not retained during data collection. As a result, standard deviations or confidence intervals could not be computed in the present study, limiting the statistical characterization of transfer performance. Future real-world evaluations will therefore record per-rollout outcomes and report variance measures to support a more complete statistical analysis. Second, the current framework still requires task-specific real-world demonstrations for encoder adaptation, which limits its applicability in settings where even a small number of demonstrations is difficult to collect. Third, simDP primarily addresses the observation and action representation gaps, but does not explicitly model dynamics mismatch, contact uncertainty, or execution noise between simulation and reality. Finally, the transfer pipeline relies on a calibrated correspondence between the simulation and real environments, including workspace scale, camera viewpoint, and end-effector coordinate conventions. Future work will focus on reducing the need for real-world supervision, extending the framework to jointly handle visual and dynamics discrepancies, and validating the method on more diverse robots, richer real-world data, and long-horizon manipulation tasks.

## Figures and Tables

**Table 1:** Real-world normalized task completion scores (0–1) on the MimicGen-derived tasks. Each value is the average over 15 real-world rollouts. The best result in each column is shown in bold.

Method	Stack Three	Three Piece Assembly	Threading	Square	Average
ACT [29]	0.77	0.37	0.67	0.40	0.55
DP [2]	0.77	0.34	0.73	0.47	0.58
FMP [28]	0.83	0.47	0.80	0.53	0.66
**simDP (ours)**	**0.93**	**0.70**	**0.93**	**0.80**	**0.84**

**Table 2:** Sim-to-real transfer comparison using normalized task completion scores (0–1). Each value is the average over 15 rollouts. Zero-shot transfer, training from scratch, and simDP are evaluated on real-world rollouts. In contrast, DP (sim-trained, sim-evaluated) denotes a diffusion policy trained and evaluated entirely in simulation; this row is included solely as a simulation-only reference point and should not be interpreted as an upper bound on real-world performance.

Training Strategy	Stack Three	Three Piece Assembly	Average
Zero-shot transfer	0.00	0.00	0.00
Training from scratch	0.77	0.34	0.56
**simDP**	**0.93**	**0.70**	**0.82**
DP evaluated on simulations	0.74	0.46	0.60

**Table 3:** Data-efficiency study with varying numbers of real-world demonstrations. Each entry is reported as the average over 15 real-world rollouts for *Stack Three*/*Three Piece Assembly*.

Real Demonstrations	10	25	50
DP trained only on real data	0.13/0.07	0.50/0.30	0.77/0.34
simDP (ours)	**0.63**/**0.43**	**0.83**/**0.67**	**0.93**/**0.70**

## Data Availability

The simulation data are available from the corresponding public sources cited in the manuscript. The real-world robot demonstration data collected for this study are available from the corresponding author upon reasonable request. The experimental videos are accessible at https://simdp.github.io/ accessed on 23 June 2026.
